# Digital Tools Designed to Obtain the History of Present Illness From Patients: Scoping Review

**DOI:** 10.2196/36074

**Published:** 2022-11-17

**Authors:** Carl T Berdahl, Andrew J Henreid, Joshua M Pevnick, Kai Zheng, Teryl K Nuckols

**Affiliations:** 1 Cedars-Sinai Medical Center Los Angeles, CA United States; 2 University of California Irvine Donald Bren School of Information and Computer Sciences Irvine, CA United States

**Keywords:** anamnesis, informatics, emergency medicine, human-computer interaction, medical history taking, mobile phone

## Abstract

**Background:**

Many medical conditions, perhaps 80% of them, can be diagnosed by taking a thorough history of present illness (HPI). However, in the clinical setting, situational factors such as interruptions and time pressure may cause interactions with patients to be brief and fragmented. One solution for improving clinicians’ ability to collect a thorough HPI and maximize efficiency and quality of care could be to use a digital tool to obtain the HPI before face-to-face evaluation by a clinician.

**Objective:**

Our objective was to identify and characterize digital tools that have been designed to obtain the HPI directly from patients or caregivers and present this information to clinicians before a face-to-face encounter. We also sought to describe outcomes reported in testing of these tools, especially those related to usability, efficiency, and quality of care.

**Methods:**

We conducted a scoping review using predefined search terms in the following databases: MEDLINE, CINAHL, PsycINFO, Web of Science, Embase, IEEE Xplore Digital Library, ACM Digital Library, and ProQuest Dissertations & Theses Global. Two reviewers screened titles and abstracts for relevance, performed full-text reviews of articles meeting the inclusion criteria, and used a pile-sorting procedure to identify distinguishing characteristics of the tools. Information describing the tools was primarily obtained from identified peer-reviewed sources; in addition, supplementary information was obtained from tool websites and through direct communications with tool creators.

**Results:**

We identified 18 tools meeting the inclusion criteria. Of these 18 tools, 14 (78%) used primarily closed-ended and multiple-choice questions, 1 (6%) used free-text input, and 3 (17%) used conversational (chatbot) style. More than half (10/18, 56%) of the tools were tailored to specific patient subpopulations; the remaining (8/18, 44%) tools did not specify a target subpopulation. Of the 18 tools, 7 (39%) included multilingual support, and 12 (67%) had the capability to transfer data directly into the electronic health record. Studies of the tools reported on various outcome measures related to usability, efficiency, and quality of care.

**Conclusions:**

The HPI tools we identified (N=18) varied greatly in their purpose and functionality. There was no consensus on how patient-generated information should be collected or presented to clinicians. Existing tools have undergone inconsistent levels of testing, with a wide variety of different outcome measures used in evaluation, including some related to usability, efficiency, and quality of care. There is substantial interest in using digital tools to obtain the HPI from patients, but the outcomes measured have been inconsistent. Future research should focus on whether using HPI tools can lead to improved patient experience and health outcomes, although surrogate end points could instead be used so long as patient safety is monitored.

## Introduction

### Background and Significance

Many medical conditions, perhaps 80% of them, can be diagnosed by taking a thorough history of present illness (HPI) [1]. However, in the clinical setting, situational factors such as interruptions and time pressure may cause interactions with patients to be brief and fragmented [2]. One solution for improving clinicians’ ability to collect a thorough HPI and maximize efficiency and quality of care could be to use a digital tool to obtain the HPI before face-to-face evaluation by a clinician.

The concept of using a computer to aid in history taking or diagnosis is not new. In fact, some clinicians entered data into computers as early as the 1940s and used software to generate differential diagnoses [3]. In the 1980s, a small minority of clinicians began asking patients to interact with computers directly to enter their own histories, but the process was cumbersome in many cases because patients had to answer dozens or even hundreds of questions [4]. In the early 2000s, investigators tried new methods to decrease the number of required questions, but such tools did not become popular—perhaps because they were not well integrated into emerging electronic health record (EHR) systems [5].

In the contemporary digital age, software developers and research groups in the health sector are developing tools to engage patients in diagnosis and management of their health problems [6-8]. Patients are becoming accustomed to collecting health-related information on their own devices and submitting it to their clinicians. Moreover, starting in 2011, the US federal government began to encourage clinicians and health care systems to collect such information, called patient-generated health data (PGHD), and use it in a meaningful manner. Examples of commonly submitted PGHD include blood pressure measurements, blood glucose measurements, and patient-reported outcome measures for chronic conditions [9,10]. Patient-generated HPI is a less ubiquitous form of PGHD but leveraging it could improve the efficiency and quality of patient care if it is done thoughtfully.

### Objectives

In this scoping review, our objective was to identify and characterize patient-facing digital tools that obtain the HPI and present it to clinicians before an in-person encounter. We also sought to describe outcomes reported in studies of these tools, especially those related to usability, efficiency, and quality of care.

## Methods

### Search Strategy

In consultation with a medical librarian, we developed search terms designed to identify HPI tools of interest from peer-reviewed sources. We then searched the following databases: MEDLINE, CINAHL, PsycINFO, Web of Science, Embase, IEEE Xplore Digital Library, ACM Digital Library, and ProQuest Dissertations & Theses Global. The search was performed in November 2019, and it included all original research and commentary articles that were available in English (for more details of the search strategy, refer to Multimedia Appendix 1).

### Article Selection

Titles and abstracts that resulted from the literature search were imported into *DistillerSR* (Evidence Partners) to facilitate screening. Two independent reviewers (CTB and AJH) evaluated all titles and abstracts for relevance based on the inclusion criteria: (1) patient-facing digital tools that obtain the HPI directly from patients or caregivers and (2) present this information to clinicians before a face-to-face encounter. Tools were excluded if they were administered by the clinician rather than the patient, designed to track symptoms over time rather than make a new diagnosis, designed to screen for only one diagnosis (eg, COVID-19 infection), or if there was no mention of outcome measures in any literature describing the tool. Next, in a full-text–review stage, both reviewers reviewed the full text to determine whether the article met the inclusion and exclusion criteria. If there was any disagreement about whether the tool should be included, it was resolved by consensus.

### Data Extraction and Synthesis

To obtain information characterizing each tool, a member of the research team (AJH) reviewed the original source material that identified the tool, any other cited references in the original source, and relevant websites of the tool identified through web searches. We developed narrative descriptions of the tools and maintained this information in a data spreadsheet along with their associated references.

To develop a taxonomy of the tools identified, 2 reviewers (CTB and AJH) used a qualitative pile-sorting method [11]. We started by writing the names of tools on small pieces of paper and arranging them into groups that were qualitatively similar. Next, we discussed what qualities the tools shared and which ones made them different from one another. Once we had compiled a list of these defining characteristics, we used them to categorize the tools in our sample.

Subsequently, we held a discussion among the research team to review characteristics of the various tools. Informed by this discussion and our newly developed taxonomy, a member of the research team (AJH) reviewed all available materials once again and performed targeted data extraction, including tool name, name of vendor or developer, availability of multilingual support, year of initial development or mention, intended patient user population (eg, pediatric, chest pain, or pulmonary), modality of query delivery (eg, narrative vs structured), decision support capability (patients, clinicians, or both), integration with clinical information systems such as EHRs (yes or no), and outcome measures used in evaluation. A second member of the research team (CTB) reviewed all available materials to verify the accuracy of extracted data. After these steps were completed, we cowrote brief narratives to describe each tool. Finally, we contacted the developer or vendor for each tool to verify the information we had collected.

**Figure 1:**
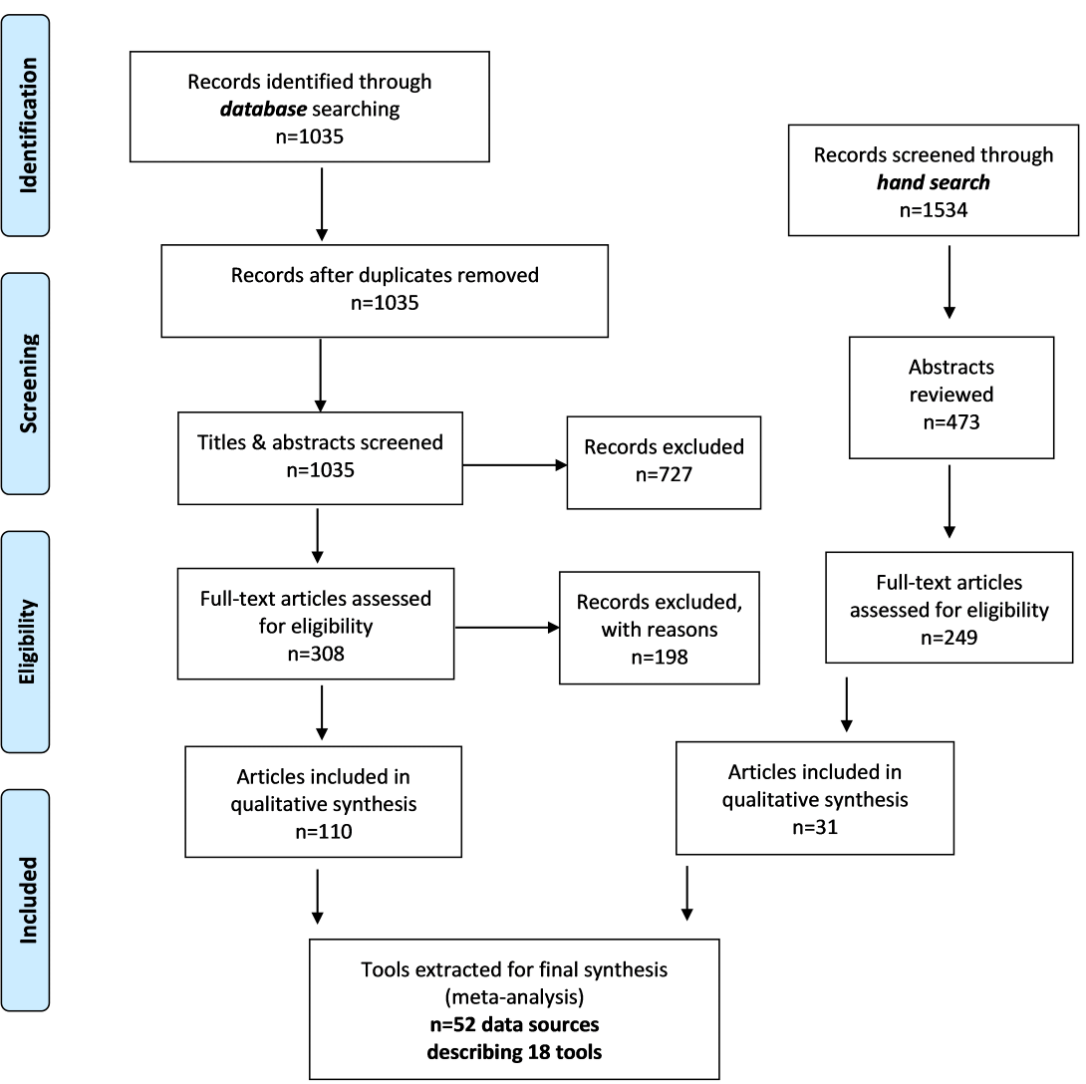
Flowchart demonstrating article inclusion and exclusion for our scoping review according to PRISMA-ScR (Preferred Reporting Items for Systematic Reviews and Meta-Analyses extension for Scoping Reviews) guidelines [12,13].

This study followed the PRISMA-ScR (Preferred Reporting Items for Systematic Reviews and Meta-Analyses extension for Scoping Reviews) guidelines for scoping reviews [12,13]. The PRISMA-ScR flow diagram is presented in Figure 1.

## Results

### Overview

Our literature search identified 2569 publications of potential interest. After duplicates were removed and titles and abstracts were screened, 557 articles underwent full-text assessment for eligibility. A total of 141 articles met inclusion criteria and were included in our qualitative synthesis. We encountered a total of 18 unique tools to include in our review, which were described by 52 data sources that included outcome measures. For a comparison of individual tools and their characteristics, refer to Table 1, We also compiled a list of synonyms for the process of obtaining the HPI from patients or caregivers using a digital tool, which may be a helpful reference for future investigators (Multimedia Appendix 2).

**Table 1:** Data extraction table for tools used to obtain the history of present illness.

Name	Developer	Year	Outcome measures	Interaction	Lingual support	Data entry	Patient subpopulation	Delivery	Decision support
Instant Medical History [14-16]	Primetime Medical Software	1985	Completion rate, completion time, and patient usability	Multiple choice	English	Patient and clinician	All	Import to EHR^a^	Clinician
HELP System [17,18]	LDS Hospital	1986	Diagnostic agreement (physician vs tool)	Multiple-choice, open-ended free text	English	Patient	Pulmonary	Import to EHR	Clinician
AIDA [19,20]	Erasmus University	1987	Patient usability, completion time, and complaint agreement and diagnostic agreement (physician vs tool)	Multiple-choice, open-ended free text	Dutch	Patient	Respiration, circulation, gastrointestinal, genitourinary, nervous system, skin, and general disorders	Text-based report	Patient and clinician
ParentLink [21,22]	Blackboard	1989	Completion rate of critical history elements (physician vs tool) and completion time	Multiple-choice, open-ended free text	English	Patient	Pediatrics, emergency, allergy, and trauma	Import to EHR	Clinician
CIDI-Auto [23-26]	Canberra Hospital (computerized version of the World Health Organization’s Composite International Diagnostic Interview)	1997	Patient acceptability and diagnostic agreement (physician vs tool)	Multiple-choice, open-ended free text	Multiple languages	Patient and clinician	Psychiatry	Text-based report	Clinician
MEDoctor [27,28]	MEDoctor Systems, Inc	1999	Diagnostic agreement (vignettes vs tool)	Multiple choice	English	Patient	All	Text-based report	Patient
Clinical Expert Operating System (CLEOS) [29,30]	Karolinska Institutet	2008	Patient satisfaction, percentage agreement of symptoms (physician vs tool), and accuracy in excluding acute coronary syndrome	Multiple choice	English, German, and Swedish	Patient	Cardiology	Import to EHR	Clinician
Mediktor [31-36]	Teckel Medical	2011	Diagnostic agreement (physician vs tool)	Conversational, multiple choice	Many languages (>180)	Patient	All	Text-based report	Patient
DocResponse [37-40]	DocResponse	2012	Diagnostic agreement (vignettes vs tool)	Multiple choice	English	Patient and clinician	All	Import to her	Patient and clinician
Digivey [41]	Creoso, in collaboration with researchers at Johns Hopkins University	2013	Patient usability, time to completion, and data entry error rate	Multiple choice	English	Patient	Emergency	Import to her	Clinician
PatientTouch [42]	PatientSafe Solutions Inc	2014	Patient usability and satisfaction	Multiple choice	English, and Spanish	Patient	Emergency	Import to EHR	Clinician
OurNotes [43-45]	OpenNotes	2015	Patient experience and clinician workload (qualitative study)	Open-ended free text	English	Patient and clinician	All	Import to EHR	None
FirstHx [46,47]	FirstHx Corp	2016	Time to completion, patient usability, and number of questions asked (physician vs tool)	Multiple choice	Multiple languages (10)	Patient	All	Import to EHR	Clinician
Automated Evaluation of Gastrointestinal Symptoms (AEGIS) [48,49]	My Total Health	2016	Rating of tool note quality (comparing physician note vs tool note) and agreement of alarm features (physician vs tool)	Multiple choice	English	Patient	Gastroenterology	Text-based report	Clinician
Digital Communication Assistance Tool (DCAT) [50-53]	aidminutes GmbH	2017	Completion time, patient and physician usability, percentage agreement in symptoms reported, and repeat clinic visits	Multiple choice	Multiple languages (21)	Patient	All	Import to EHR	Clinician
Quro [54-56]	Medius Health	2017	Diagnostic agreement (vignette vs tool)	Conversational, open-ended free text	Multiple languages	Patient	All	Text-based report	Patient
Mandy [57]	Precision Driven Health	2017	Diagnostic agreement (vignette vs tool)	Conversational, open-ended free text	English	Patient	All	Import to EHR	Clinician
Diagnosis and Anamnesis Automated Medical History–Taking Device (DIAANA AMHTD) [58]	Logic-Based Medicine Sàrl, in collaboration with Lausanne University Hospital	2019	Diagnostic agreement (physician vs tool)	Multiple choice	German	Patient	Musculoskeletal	Import to EHR	Clinician

^a^EHR: electronic health record.

### Narrative Descriptions of Tools

As the tools we identified differed in many ways (eg, stated purpose, intended setting of use, and outcome measures), we developed a narrative description of each tool (Textbox 1).

**Narrative descriptions of the tools used to obtain the history of present illness from patients.**

**Digital tools used to obtain the history of present illness and their descriptions**

- Instant Medical History
  General description: Instant Medical History is a tool that was developed by Primetime Medical Software in 1985 to obtain comprehensive information about the history of present illness while also saving physician time and making documentation more complete. The tool has evolved over the last several decades, and it is still in use today.
  Design: patients are invited to select a chief complaint through a web-based portal from home or in a medical office waiting room. They are then presented with a multiple-choice–question set about their symptom severity, duration, timing, context, modifying factors, and associated signs of illness. This information is next submitted to the electronic health record through an application programming interface for review before the patient visit and additionally for inclusion in the physician’s note, if desired [14].
  Outcomes measured: the company reports that the tool may save up to 6 minutes per clinical encounter [15,16].
  Extent of use: the tool is currently being used in 7 countries by 44,500 physicians. The vendor estimates that it will be used in 80 million visits in 2020 (email communication with Matthew Ferrante, Primetime Medical Software, July 21, 2020).

- HELP System
  General description: the HELP System, programmed on the Microsoft Query driver, was described in a 1987 publication by Haug et al [17] titled “A Decision-Driven System to Collect the Patient History.” Informaticists at the University of Utah described a computer-administered history-taking system with decision-driven questions designed to create a differential diagnosis for hospital inpatients with pulmonary disease.
  Design: the system used a cognitive model of question selection along with a Bayesian scoring algorithm that led to targeted question selection using modular diagnostic frames within a program called QUERY. The program contained yes-or-no questions for up to 182 symptoms; however, using the decision-driven system, patients in the study were asked to answer a mean of 51 (SD 31) questions. The program’s response report was a list of top 5 differential diagnoses, with accompanying likelihood ranging from 0 to 1.
  Outcomes measured: when compared with documented discharge diagnoses in a sample of 27 study participants, the tool’s list of 5 differential diagnoses included the principal discharge diagnosis for 85% of the patients.
  Extent of use: the tool was also integrated into the hospital’s HELP hospital information system, which pioneered clinical decision support. A subsequent set of tests with an updated diagnostic system and a modified approach to questioning was tested later and reported in the American Association for Medical Systems and Informatics proceedings, including several refinements to the data collection process. Although the tool is no longer in use within the hospital setting, it formed the basis for a subsequent diagnostic application, Iliad, that has been used in medical education [18].

- AIDA
  General description: AIDA is a software package developed by the department of medical informatics at Erasmus University, Rotterdam, The Netherlands. Its capability to automate medical history taking was described in 1987 by Quaak et al [19] in a special issue of *Computer Methods and Programs in Biomedicine*. The tool’s stated purpose was to elicit a comprehensive history and aid physicians in arriving at an accurate diagnosis.
  Design: patients were asked to read questions on a screen and press keys corresponding with their answers. The system contained >400 questions relating to 179 different items. Regarding acute complaints, the patient was asked system by system about whether symptoms existed (using a 7-point scale from *never* to *always*). If the patient indicated the presence of a symptom, the system asked more questions about frequency, severity, intensity, duration, onset, and location. The final report was displayed to physicians and patients in a narrative format that was designed to mirror how physicians wrote notes.
  Outcomes measured: the investigators studied the tool’s performance compared with the gold standard of a physician interview and found agreement to be 25%. They ultimately found that the tool led to a higher number of *diagnostic hypotheses* and higher diagnostic certainty compared with physician interview alone. Of note, patients required an average of 66 minutes to complete the computerized interview, but their reports of the experience were favorable (92% rated the tool *useful*) [20].
  Extent of use: the tool was never further developed into a product used in routine care.

- ParentLink
  General description: ParentLink is a tool designed to obtain information from parents to describe their children’s symptoms in the pediatric emergency department setting. ParentLink, the company, was founded in 1989 and acquired by Blackboard in 2014, and the earliest clinical publication was authored by Porter [21] of Boston Children’s Hospital in 1999.
  Design: parents accompanying children with nontraumatic complaints were invited to submit data describing the history of present illness. Parents were asked to independently answer questions on an electronic terminal regarding structured question pathways for fever, respiratory symptoms, or gastrointestinal symptoms. If the reason for the visit was not listed, the parent was invited to select *other*, and they were then shown a textbox for entering open-ended free text. For structured question pathways, parents were invited to answer prompts such as their children’s activity level, fluid intake, and urine output.
  Outcomes measured: parental data entry took a mean time of 5 minutes, and data were not shown to treating physicians. The investigators measured the validity of content entered by parents and found it to be *comparable* with information documented by physicians with improved sensitivity of parental documentation for hydration status. In a related study from 2002 characterizing the free-response pathway, parents entered between 1 and 142 words to describe the reason for the visit (when presented with a character limit of 2048, which is approximately 350-400 words). Most parents described the chief complaint and elements of the history of present illness, and some asked specific questions and added information about the past medical history. When the parents’ text input and physicians’ histories in the electronic health record were compared, 23% (7/30) of the parents’ entries noted details or observations that were not documented by the physicians [21].
  Extent of use: subsequent studies of ParentLink have included pediatric patients with other complaints, including head trauma, ear pain, and dysuria [22].

- CIDI-Auto
  General description: CIDI-Auto is a computerized version of the World Health Organization’s Composite International Diagnostic Interview (CIDI). The tool’s intended purpose is to allow patients to privately answer a series of questions that lead to the automatic generation of a list of psychiatric differential diagnoses. The computerized version was first described and evaluated by investigators at Canberra Hospital in Canberra, Australia, in 1997 [23].
  Design: patients were asked to sit at a computer workstation during an acute psychiatric hospital admission and answer yes-or-no questions about psychiatric symptoms. The core module of the instrument (CIDI-Core) contained 20 major questions and 59 subquestions, which took approximately 75 minutes to administer [24]. Each patient’s responses were organized into a report of diagnosis and symptoms that was given to a physician. The report consisted of a summary of active International Classification of Diseases, Tenth Revision, diagnoses (active in the last 30 days); lifetime diagnoses, which were active >1 month ago; and symptoms in major diagnostic areas.
  Outcomes measured: psychiatric physicians (the *gold standard*) agreed with 50% of the CIDI-Auto current diagnoses and indicated that only 22% of the CIDI-Auto reports provided useful new diagnoses, although 63% helped to clarify diagnoses, and 58% could save clinicians some time. They endorsed the CIDI-Auto as a possible aid to indirect or remote diagnosis where histories would be taken by nonexpert staff. With regard to patients, 94% liked the computerized interview, 83% understood the questions without difficulty, and 60% felt more comfortable with the computerized interview than with a physician. Education and previous computer experience promoted positive attitudes and satisfaction with the computerized interview [25].
  Extent of use: the CIDI-Auto has now evolved into the World Health Organization World Mental Health-CIDI Instrument, which is now administered by computer in diverse settings across the world [26].

- MEDoctor
  General description: MEDoctor is a commercially produced symptom checker designed to produce a list of differential diagnoses for users with acute or chronic symptoms [27]. MEDoctor Systems Inc, the developer of the tool, was founded in 1999, and the company’s renewed mission statement since 2017 defines its goal as providing patients access to “actionable medical information...before seeing the clinician” so that patients can make cost-effective decisions about their health.
  Design: the MEDoctor tool uses a *rule out* basis that includes the value of negative symptom answers (*no input*) to rule out disease probabilities. The engine navigates through >4200 symptoms using Bayesian statistics to produce each interview item based upon numerous factors accumulated, such as sex, onset, and yes-or-no responses. The patient interface consists of drop-down menus and yes-or-no questions to characterize their symptoms. At the end of the process, patients are presented with a list of the top 3 differential diagnoses, and they are offered an opportunity to view a text-based report that displays all completed responses and can be sent to a clinician [28].
  Outcomes measured: diagnostic agreement between the tool and vignettes is measured.
  Extent of use: The diagnostic tool has been used worldwide for >5 years. As of July 2020, according to the chief executive officer, it has been completed 36,860 times by users in the United States, the United Kingdom, South Africa, and the Philippines (email communication with Charles Kelly, MEDoctor Systems Inc, July 21, 2020).

- Clinical Expert Operating System
  General description: Clinical Expert Operating System (CLEOS) is a tool created in 2008 by Zakim [29] that is currently being tested in a clinical trial at Karolinska Institutet in Stockholm, Sweden. The tool is designed to facilitate thorough history taking from patients, and it also includes capabilities for decision support of diagnosis, management, and risk stratification for clinicians.
  Design: the history-taking program is based on the principles of pathophysiology formalized as software algorithms representing medical knowledge as 450 decision trees. A gating mechanism plus feed-forward and feedback loops enable the tool to perform detailed explorations of any aspects of the history that are significant while also avoiding issues of medical redundancy. The data obtained via CLEOS can be formatted into a narrative summary of the most pertinent history findings similar to that of a physician’s note.
  Outcomes measured: in a study of 45 patients published in 2008 in which patients underwent usual care and completed a CLEOS interview, the tool detected 3.5 additional problems per patient, some of which were deemed to be clinically significant, such as unrecognized transient ischemic attacks [29]. Currently, the tool is being used in a clinical trial at Karolinska University Hospital in Stockholm, Sweden, for evaluation of patients with chest pain. After initial triage by a physician in the emergency department, patients are invited to answer questions within CLEOS on a tablet computer. Data about pain in the context of the differential diagnoses for chest pain are contained within 29 decision graphs presenting questions that are a mixture of yes-or-no, multiple-choice, and image-based question types. The arrangement of questions for these patients is based on principles of cardiovascular pathophysiology and includes ratings of symptom severity, nature, pain location and radiation, associated cardiac or vagal symptoms, and precipitators of pain. The primary outcome measure is the successful exclusion of acute coronary syndrome at 7 days (using physician diagnosis as the comparator). Secondary outcomes include the ability to calculate risk scores for acute coronary syndrome; exclusion of acute coronary syndrome for 30 days and 1 year; direct costs and resource use; and patient experience regarding feasibility, acceptance, comprehensibility, and technical aspects such as usability [30].
  Extent of use: in the clinical trial, CLEOS is being used at a single hospital: Danderyd University Hospital in Stockholm, Sweden.

- Mediktor
  General description: Mediktor is a symptom-checker tool developed in 2011 by Teckel Medical and StartUp Health, an IBM affiliate. The developers describe Mediktor as “an interactive tool that can analyze users’ symptoms and evaluate their state of health” [31]. Mediktor is available for consumers to use for free on its website as well as major smartphone and tablet systems in the form of a downloadable mobile app [32,33].
  Design: the tool presents users with either multiple-choice questions or conversational-style prompts. After a series of questions and responses, users are provided with an assessment of triage urgency, a summary sheet denoting inputted responses and possible diagnoses, and an opportunity to pay for a telemedicine visit with a licensed clinician. Accessible to patients in >180 supported languages, the tool is powered by artificial intelligence and natural language processing.
  Outcomes measured: according to an academic study conducted in Spain, Mediktor’s diagnostic accuracy was tested against the gold standard of physician diagnosis, and its primary list of diagnoses matched in 91% of the cases [36].
  Extent of use: the tool has become available through Amazon’s Alexa as a skill that can be enabled for free [34] and through Telegram as a chatbot messaging feature [35]. According to the developer, the tool is currently being used at 3 clinical sites in Europe and the United States with 1.2 million user evaluations in 2019 (email communication with Fabiana Rojas, Mediktor, July 21, 2020).

- DocResponse
  General description: DocResponse is a clinical workflow, patient intake, and documentation tool launched in 2012 that engages users electronically to schedule visits and enter previsit data about past medical history and acute complaints before their face-to-face encounter with a clinician. The tool was developed by a team of technology experts and physicians from various specialties [37]. The tool’s stated goal is to reduce data entry by front desk reception personnel, medical assistants, and clinicians.
  Design: patients are invited to enter data on any hospital-provided or personal smart device. The user can complete consent forms; enter data describing past medical, family, surgical, and social histories; complete the review of systems; and use an *assessment tool* that populates the history of present illness in the medical record before the patient’s arrival. Clinicians are then provided with clinical decision support, including a preliminary differential diagnosis and treatment recommendation. At the end of the visit, the tool can also generate relevant education materials for the patient to review [38].
  Outcomes measured: in a 2015 study, DocResponse was found to be the symptom checker most likely to arrive at the correct principal diagnosis out of a sample of 23 similar tools (although its diagnostic accuracy was 18 out of 36 [50% CI 33%-67%]) [39,40].
  Extent of use: the tool is currently being used at >170 clinical sites in many care settings such as urgent care, primary care, orthopedics, pediatrics, gastroenterology, multispecialty, and federally qualified health centers throughout the United States. Per vendor report, the tool was used for >225,000 encounters in 2019 (email communication with Tarek Fahl, MD, DocResponse, July 16, 2020).

- Digivey
  General description: Digivey is a self-administered computer-assisted interview tool (delivered on Digivey survey software) that was designed to improve diagnostic accuracy and patient safety.
  Design: A 2013 academic publication from Newman-Toker’s research group at Johns Hopkins University School of Medicine described the design of this tool [41]. Digivey delivered adaptive questionnaires with approximately 40 items to emergency department patients to elicit clinical history information about their individual symptom presentations. Participants used one of three electronic devices: mobile kiosk, touch-screen monitor, or laptop computer.
  Outcomes measured: all 3 electronic device interfaces were deemed to be usable, there were low rates of user error, and administration required approximately 6 minutes [41].
  Extent of use: Newman-Toker has continued to use the system extensively in research studies, including for data collection from patients in an ongoing 5-site multicenter clinical trial that screened >3000 patient encounters using the tool. The Johns Hopkins department of neurology is working to deploy the system in several clinical areas, including via mobile platforms where patients will be able to consistently self-administer their medical data (email communication with David Newman-Toker, MD, July 7, 2020).

- PatientTouch
  General description: PatientTouch is a tool created by PatientSafe Solutions Inc to elicit patients to use an electronic questionnaire to describe their symptoms. In a pilot study published in 2014, investigators at Los Angeles County+USC Medical Center described the tool’s use among adult medically stable patients presenting to the emergency department.
  Design: patients were asked to use a handheld touch-screen tablet and complete an electronic questionnaire in either English or Spanish before contact with their physician. First, eligible patients were prompted to select one of six chief complaints: low back pain, upper extremity injury, lower extremity injury, abdominal pain, headache, or motor vehicle collision. Next, they were guided through chief complaint–specific algorithms based in questions provided in multiple-choice or point-and-click format.
  Outcomes measured: users were asked to rate their experience, usability, and satisfaction with the technology. Patients reported feeling that the device would help them better communicate with their physician and improve their overall quality of care [42].
  Extent of use: the tool is not presently being used at any clinical sites, according to its developer.

- OurNotes
  General description: OurNotes is a tool developed in 2015 by the OpenNotes movement in which the patient is invited to contribute information to their own ambulatory visit notes [43]. The developers’ stated goal is to enhance patient-clinician communication, support patients’ engagement in their care, and save clinician time.
  Design: a pilot study publication described the enrollment of primary care clinicians and patients who were already registered to use an institutional messaging portal. A few days before a scheduled visit, the patient was asked to submit answers to two open-ended free-text questions: “How have you been since your last visit?” “What are the most important things you would like to discuss at your visit?” Before the visit, the patient’s answers to these inquiries were routed to the clinician who could then incorporate them into the visit note. The patient could view the note by logging in to the patient portal after the visit [44].
  Outcomes measured: patient experience and clinician workload were assessed in a qualitative study, with patients *supporting the idea* and clinicians thinking it was possible that their workload could decrease if patients helped to produce their own visit notes [44].
  Extent of use: 4 academic medical centers participated in the pilot study, with 160 primary care clinicians and 2500 patients participating between 2018 and 2020. Evaluation of the pilot is underway, and pilot sites are considering expansions of the program [45].

- FirstHx
  General description: FirstHx is a patient intake tool founded in 2016 and developed by physicians in Toronto, Ontario, Canada, that collects information describing a patient’s symptoms and generates focused medical histories in advance of an acute care visit. The developer’s stated goals include a dedication to improving physician-patient communication, reducing documentation time, mitigating medical error, and improving the quality of care [46].
  Design: it is built specifically for use in emergency departments, urgent care, and telemedicine. The tool is designed to use a line of questioning similar to that of physicians and generate a history of present illness report covering >240 presenting complaints in up to 10 supported patient languages.
  Outcomes measured: the company’s website reports that patients can complete the 3- to 6-minute digital intake using either their personal smartphone, provided tablet device, or through a dedicated kiosk [47].
  Extent of use: the developers state that it is available in the Epic App Store. It has undergone pilot testing at >10 sites, and its estimated use will be 600,000 visits per year (email communication with Mark Benaroia, MD, FirstHx, January 27, 2022).

- Automated Evaluation of Gastrointestinal Symptoms
  General description: Automated Evaluation of Gastrointestinal Symptoms (AEGIS) is a tool developed in 2016 by researchers in Los Angeles, California, United States, and Ann Arbor, Michigan, United States, to automatically obtain reports of symptoms from patients in the gastroenterology clinic and transform them into a coherent history of present illness.
  Design: patients are invited to answer questions through a web-based portal to characterize symptoms as delineated by the Patient-Reported Outcomes Measurement Information System framework. If a patient reports several symptoms, the AEGIS system prompts the user to select the most bothersome symptom. An algorithm generates a physician-facing report that is designed to look like a physician-generated history of present illness.
  Outcomes measured: in 1 peer-reviewed publication, patients underwent both computer-generated history taking and usual care, and blinded ratings compared the quality of both sets of documentation. Computer-generated histories were found to be more complete, more useful, better organized, more succinct, and more comprehensible [48]. Another peer-reviewed publication focused on the AEGIS system’s ability to detect alarm features and found its performance to be superior to physician detection (alarm features detected in 53% vs 27%, respectively) [49].
  Extent of use: AEGIS has not been used in routine patient care outside of the aforementioned studies (email communication with Christopher Almario, MD, Cedars-Sinai Medical Center, July 19, 2020).

- DCAT
  General description: DCAT, created in 2017 by German research and technology experts at *aidminutes GmbH*, is an anamnesis tool that facilitates communication between patients and health care providers in primary care settings, with particular attention to refugee care sites in Germany that experienced an influx of Syrian patients. Aidminutes, which is affiliated with the department of general practice at the University Medical Center Göttingen in Göttingen, Germany, refers to the tool as a digital communication assistance tool that aims to improve diagnostic accuracy by improving medical history taking [50].
  Design: once patients arrive at the outpatient clinic waiting room, they are given tablet devices that allow them to enter information describing their symptoms. The tool also facilitates entry of data describing past medical history, current medications, allergies, and any psychological comorbid conditions. To facilitate use by patients with limited literacy, the tool is designed to be visually intuitive, and it also includes audio prompts. After patients enter their data, responses are translated into the clinician’s preferred native language and presented as a data synopsis, including alerts regarding *red flags* in the history that have been discovered [51].
  Outcomes measured: use of the tool has been described in peer-reviewed publications, including a recent study demonstrating good usability and acceptance by patients speaking Levantine Arabic, Modern Standard Arabic, Egyptian Arabic, Farsi, Sorani Kurdish, and Turkish [52]. In 1 study, patients successfully completed their assessments in an average time of 13 minutes [53].
  Extent of use: the tool was used in approximately 10,000 multilingual visits in urgent care and family medicine clinics during 2021 (email communication with Frank Muller and Andreas Lippke, May 3, 2022).

- Quro
  General description: Quro is a *chatbot health assistant* created in 2017 by Medius Health (Sydney, New South Wales, Australia) that uses machine learning artificial intelligence to deliver health assessments to users [54]. Patients can access the tool through a desktop web browser or smartphone.
  Design: users are invited to answer a set of free-response and multiple-choice prompts generated by the back-end sequential question prediction algorithm using a large-scale clinical knowledge graph to mimic the taking of a medical history. Each new question is predicted based on previous user-chat context. After completion, the patient is provided a list of differential diagnoses with interpretations, nearby health services, recommendations about the urgency of their condition, and a detailed report that displays the answers to their individual set of questions. The tool’s website advertises a built-in medical dictionary of >7 million disease and illness presentation patterns, scored against content sourced from “trusted sources like medical journals” to produce its personalized clinical assessments [55].
  Outcomes measured: in an article published by Quro’s developers, the tool’s triage accuracy was assessed using 30 case-based scenarios (10 for emergency care, 10 for general practitioner care, and 10 for self-care) and found to be accurate in 83% (25/30) of the cases [56].
  Extent of use: Quro is marketed to, and used by, several health and wellness service providers to engage with, and onboard, patients remotely (email communication with Shameek Ghosh, founder and chief technology officer, September 1, 2020).

- Mandy
  General description: Mandy is a primary care conversational-style dialogue system developed in 2017 by a public-private research partnership of computer scientists at the University of Auckland in Auckland, New Zealand, funded by Precision Driven Health and Orion Health, aimed at improving health outcomes through data science. The tool is designed to assist health care staff by automating the patient intake process.
  Design: patients interact with the tool by answering the conversational-style prompts with open-ended free-text responses. An analytic engine uses natural language processing to interpret the patient’s text, queries a symptom-to-cause mapper for reasoning about potential diagnostic causes, and then generates further interview questions. Once the system has obtained sufficient information from the patient, it reports a differential diagnosis for the clinician to consider.
  Outcomes measured: in a proof-of-concept paper, the developers reported on the application’s *question accuracy* (ability to generate key follow-up questions after an initial chief complaint) and diagnosis *prediction accuracy* (ability to generate relevant differential diagnoses after obtaining responses to its questions) by using gold-standard cases from a medical textbook. Out of 6 cases, the tool generated appropriate questions in 5 cases and had case-by-case prediction accuracy ranging from 14% to 100% [57].

- Diagnosis and Anamnesis Automated Medical History–Taking Device
  General description: Diagnosis and Anamnesis Automated Medical History–Taking Device (DIAANA AMHTD) was developed by Logic-Based Medicine Sàrl in collaboration with Adrien Schwitzguebel of Lausanne University Hospital in Lausanne, Switzerland, to improve diagnostic accuracy through helping physicians to generate more comprehensive differential diagnoses. To date, the tool exclusively addresses musculoskeletal complaints.
  Design: in a pilot study published in 2019, the developer and coinvestigators tested DIAANA AMHTD at a teaching hospital in Geneva, Switzerland. Patients were eligible if they were waiting to be seen at an ambulatory clinic for evaluation of musculoskeletal symptoms. Patients in the experimental group were asked to complete a digital form on a touch pad before the resident physician’s evaluation, including questions about specific symptoms and risk factors. Through the completion of an adaptive questionnaire that draws from a data set of 269 questions, DIAANA AMHTD then generated a comprehensive anamnesis summary and a list of top differential diagnoses. The list of differential diagnoses (selected from 126 possibilities) was then presented to medical residents for consideration before the face-to-face evaluation.
  Outcomes measured: residents who used the tool were found to be more likely to have included the final diagnosis from the list of initial differential diagnoses than those residents who did not use the tool (75% vs 59%, respectively) [58].
  Extent of use: the tool is currently being used regularly by a single physician with 250 patient encounters in 2019 and has also been implemented by a Swiss telemedicine system called Soignez-Moi in Biel, Switzerland (Email communication with Adrien Schwitzguebel, MD, July 2020).

### Descriptions of Tool Characteristics in the Taxonomy

As a result of our pile-sorting procedure, we developed an HPI tool taxonomy that includes the following categories: interaction modality, lingual support, patient versus caregiver data entry, patient subpopulation (by age, chief complaint, or body system), modality of result delivery, and decision support target (patient or clinician). The resulting taxonomy is reported in Textbox 2.

**Taxonomy of characteristics describing the tools used for obtaining the history of present illness in the study sample.**

**Query style**

- Open-ended free text
- Multiple choice
- Conversational style (*chatbot*)

**Language capability**

- Single language only
- Multiple languages
- Discordant language support

**Tasked to perform data entry**

- Patient
- Parent or caregiver

**Patient subpopulation**

- All patients
- Limited by patient age, care setting, or body system

**Output format**

- Text-based report
- Data imported to electronic health record

**Decision support**

- Patient facing
- Clinician facing

#### Interaction Modality

Among the 18 tools reviewed, 1 (6%) used exclusively open-ended free-text interaction (OurNotes [43]), whereas 3 (17%) used a conversational style of interaction (ie, a *chatbot* style) simulating a human text-message interaction (Mediktor [31], Quro [55], and Mandy [57]). The remaining tools (14/18, 78%) used either an entirely multiple-choice format or used primarily a multiple-choice format with some open-ended free-text components.

#### Lingual Support

Of the 18 tools, 1 (6%) was developed specifically to address language barriers in Germany, and it was deployed especially to assist Syrian refugees with communication (DCAT [50]), whereas 1 (6%) specifically reported the capability to facilitate clinical communication between patients and clinicians in language-discordant encounters (FirstHx [46]); 5 (28%) other tools reported the ability to capture the HPI in English as well as other languages (CIDI-Auto [25], Clinical Expert Operating System (CLEOS) [30], Mediktor [31], PatientTouch [42], and Quro [55]), 1 (6%) was Dutch only (AIDA [19]), and 1 (6%) was German only (Diagnosis and Anamnesis Automated Medical History–Taking Device [DIAANA AMHTD] [58]). The remaining tools (9/18, 50%) were English only [16,20,21,27,40,41,46,51,57].

#### Patient Subpopulation

Of the 18 tools, 10 (56%) were tailored to specific patient populations by reason for visit or body system (HELP System: pulmonary [20]; AIDA: multiple specific body systems [19]; ParentLink: pediatric emergency, allergy, and trauma [21,22]; CIDI-Auto: psychiatry [26]; CLEOS: cardiology [29]; Digivey: emergency [41]; PatientTouch: emergency [42]; Automated Evaluation of Gastrointestinal Symptoms (AEGIS): gastroenterology [51]; and DIAANA AMHTD: musculoskeletal [58]). The remaining tools (8/18, 44%) did not specify a target subpopulation of patients [14,27,31,40,46,49,53,56,57].

#### Modality of Delivery

Of the 18 tools, 12 (67%) reported the capacity to import patient data directly into the clinician’s EHR system [14,20,21,29,40-42,46,49,53,57,58], and the other 6 (33%) provided users a text-based report of patients’ responses to prompts or a list of top differential diagnoses [19,26,27,34,51,56]. It is possible that some of the tools with text-based reports have back-end functionality that allows for transmission of information to partnering clinicians, although we are unable to ascertain which tools can do this based on review of tool web sites and articles in our sample.

#### Decision Support

Of the 18 tools, 5 (28%) offered patient-facing decision support that came either in the form of a triage acuity level or a list of differential diagnoses that was displayed for patients to read [19,27,34,40,56], whereas 15 (83%) offered clinician-facing decision support, which was displayed to clinicians as a list of differential diagnoses with or without links to evidence-based management recommendations [14,19-21,26,29,40-42,49,51,53,57,58].

### Outcome Measures

There was a wide range of outcome measures reported among the tools in the sample. Outcome measures were categorized as being related to the domains of tool usability, efficiency of care, and quality of care.

#### Tool Usability

Patient usability was studied for 33% (6/18) of the tools (Instant Medical History [14], AIDA [19], Digivey [41], PatientTouch [42], FirstHx [49], and DCAT [53]). Other related constructs were measured for several (5/18, 28%) of the other tools, including patient acceptability (CIDI-Auto [23-26]), patient satisfaction (PatientTouch [42] and CLEOS [29]), and patient experience (CLEOS [29] and OurNotes [46]). Completion rate was studied for 11% (2/18) of the tools (Instant Medical History [14] and AIDA [19]), and data entry error rate was studied for 6% (1/18) of the tools (Digivey [41]). Clinician usability was only reported for 6% (1/18) of the tools (DCAT [50,51,53,59]), although a concern about clinician workload was mentioned in the qualitative study of OurNotes [44].

#### Efficiency of Care

Time to completion was the most commonly reported measure of efficiency, used in studies of 33% (6/18) of the tools (Instant Medical History [14], AIDA [19,20], ParentLink [21,22], Digivey [41], FirstHx [46,47], and DCAT [50,51,53,59]). A study of FirstHx compared the number of questions asked by the tool and the clinician. There was no report of any research related to the impact on length of the visit or time spent by clinicians on direct or indirect care [46,47]. The protocol describing a currently ongoing clinical trial of CLEOS reported a plan to measure direct costs and resource use for patients in the intervention group versus a control group (refer to the next section [Quality of Care] for additional details) [30]. The protocol of another clinical trial reported a plan to measure the rate of repeat visits to a clinic as a proxy measure for improved patient-clinician communication at a refugee clinic (DCAT) [53].

#### Quality of Care

There were a wide variety of measures that have implications for quality of care. A study of 6% (1/18) of the tools reported agreement of the patient’s chief complaint between the tool and a physician (AIDA) [20]. Another study reported agreement in the questions asked (FirstHx) [47], and articles describing 11% (2/18) of the tools reported agreement in the symptoms reported between the tool and those the physician documented (CLEOS [30] and DCAT [53]). A study of AEGIS reported agreement in alarm features reported via the tool or in physician documentation [49], and a study of ParentLink reported completion rate of critical history elements for histories acquired via the tool and a physician [21]. Another study of AEGIS compared the quality of documentation produced by the tool with that produced by treating physicians by having blinded physicians rate the quality of documentation produced by both sources [48].

Diagnostic agreement was the most commonly reported measure related to quality of care. Studies of 22% (4/18) of the tools reported on agreement between tool-produced diagnoses and vignettes with prespecified diagnoses (MEdoctor [28], DocResponse [39,40], Quro [56], and Mandy [57]). Studies of 28% (5/18) of the tools reported agreement between physician-generated diagnoses in health care environments and diagnoses generated by the tools (HELP System [17], AIDA [19], CIDI-Auto [25], Mediktor [36], and DIAANA-AMHTD [58]). The research protocol for an ongoing study of CLEOS was the only article describing measurement of patients’ health outcomes. In the study, the primary outcome was reported to be the tool’s ability to exclude acute coronary syndrome at 7 days for patients presenting to the emergency department for chest pain, and secondary outcomes were the ability to exclude acute coronary syndrome at 30 days and 1 year [30].

## Discussion

### Principal Findings

In this scoping review, we identified 18 digital tools that have been used to collect HPI information about patients’ symptoms and communicate it to clinicians. These tools varied widely in their stated purposes (eg, to improve patient-clinician communication, to enhance patient engagement, to save clinician time, to improve diagnostic accuracy, and to exclude acute coronary syndrome). They also varied widely in their interaction modality (open-ended free text vs multiple choice vs conversational style), modality of results delivery (text-based report vs EHR integration), and decision support capability (patient facing vs clinician facing). There did not seem to be any consensus on how information should be collected or how it should be presented to patients or clinicians.

For the tools identified in this review, peer-reviewed publications describing empirical evaluation findings were somewhat limited. However, results from several studies provide preliminary evidence that implementation of such tools is acceptable to patients; for example, a well-designed usability study demonstrated that patients found the precursor to Digivey easy to use [41]; a multi-institutional survey revealed that 2 out of 3 clinicians who had used OurNotes supported continued coauthorship of visit notes with patients [45]; and studies of ParentLink and AEGIS found that both tools improved documentation of critical elements of patients’ histories [21,22,49,60]. In the coming years, investigators in Europe will determine, through a clinical trial of CLEOS, whether use of the tool can predict adverse clinical outcomes more accurately than clinicians among a sample of 2000 patients evaluated for acute chest pain. As these tools are studied more intensively, we recommend that academic evaluators should adopt one of several recently published frameworks to design comparative effectiveness studies of multiple tools [61-63]. However, although the rigorous randomized controlled trial of CLEOS is underway, other investigators and tool developers may not be willing to spend the time and resources to follow patients through the many steps in their journeys and measure health outcomes in a controlled fashion. Instead, surrogate outcomes may be considered so long as measures are in place to ensure that patients are not being harmed by HPI-tool implementation. In the emergency department work environment, for example, reducing documentation time for clinicians and improving satisfaction for patients could be adequate primary outcomes so long as there is no evidence of health outcome inferiority, such as increased length of stay for inpatients or increased return emergency department visits for discharged patients. Future investigators should consider prioritizing the following measures for patients: usability, documentation time, accuracy of the history, and satisfaction with the visit. For clinicians, we recommend prioritizing usability, documentation time, accuracy and completeness of the history (including elicitation of red flags for certain chief complaints), and satisfaction with the visit. For the visit overall, measures could include patient-clinician interaction time and emergency department length of stay (measured from the time the clinician signs up for the patient until the disposition decision).

Although academic investigators are carrying out such studies to test HPI tools, vendors of commercial products are already implementing their products in clinical environments across the world; for example, the vendor of Instant Medical History reported that it would be used in an estimated 80 million visits in 2020. To our knowledge, this tool has not been rigorously studied in peer-reviewed literature; however, it has been in use for decades, and its recent, rapid real-world implementation may indeed lead to important advances in the field of informatics.

On the basis of our review of the literature and our experience in the field, there are several barriers to the adoption of digital HPI tools by clinicians and patients. First, selecting 1 tool for testing and implementation is nontrivial because there is a lack of consensus on which features would make care more efficient and higher quality. Second, clinicians may not be interested in adopting new technology before a careful study of workflow is undertaken. Third, integration of a tool into a clinician’s EHR system may require substantial time and effort. Fourth, clinicians may fear that storing patient-generated information may increase the risk of malpractice litigation. Fifth, asking patients to use technology may exacerbate already existing health disparities for vulnerable patients. Sixth, and last, there is no clear evidence that adopting digital HPI tools leads to improved health outcomes—although evidence will likely be forthcoming in the next few years.

After evaluating all the tools described in this review, our opinion is that patients can successfully and safely be engaged to compose their own HPI information. We believe that both patients and clinicians would benefit from a tool with an intuitive design that allows patients to create a history through a combination of unstructured and structured prompts and then transforms this history into a narrative that is cohesive and communicates the key elements of the history to clinicians so that the differential diagnosis can be narrowed appropriately. We believe that the ideal tool would begin with a patient-generated narrative and then ask patients to complete a sequence of closed-ended questions to narrow the differential diagnosis. This is the sequence that clinicians are trained to use because patients can offer information that they believe is relevant, and then clinicians can use closed-ended questions to fill in gaps in knowledge and narrow the differential diagnosis [64]. For blocks of text generated by patients, natural language processing can be used to add structure [65] and feed analyses that can determine which closed-ended questions should be administered to obtain a complete history in a reasonable amount of time. This approach is similar to the one used by Quro, and it avoids the need for extremely long question chains that were used in older rule-based tools such as AIDA. This text-based approach could additionally be supplemented by touch-based graphical items so that patients would be able to signal the location of their symptoms [66]. Alternatively, it is possible that rule-based decision trees could be adequate for certain conditions in certain clinical settings. In the emergency department setting in particular, patients can present with any complaint, which makes flexibility essential for any HPI tool.

### Limitations

There are several limitations to this study. First, although we developed our search terms in consultation with a medical librarian and among a multidisciplinary research team, it is possible that the failure to include certain search terms or databases could have led us to miss key publications. Second, tools such as those we have reviewed tend to evolve over time, which may have limited our ability to characterize them accurately based on the published literature. Third, and last, our review of tools included several sources of information such as peer-reviewed literature, non–peer-reviewed articles, and websites of for-profit entities. Although we have tried to verify the accuracy of the information we obtained, it is possible that information from some sources is biased.

### Conclusions

Many HPI tools with various features are available to aid in obtaining the HPI. Future research should examine which tools improve patients’ health outcomes and which design features are pivotal to improving communication, diagnosis, and, subsequently, patient health outcomes. We recommend that future tools use a combination of narrative text, closed-ended questions, and graphical items so that the histories obtained can successfully communicate the patients’ symptoms to clinicians and narrow the differential diagnosis.

## Appendix

Multimedia Appendix 1 Search strategy.

Multimedia Appendix 2 List of synonyms for digital tools designed to obtain the history of present illness from patients.

## Abbreviations

AEGIS: Automated Evaluation of Gastrointestinal Symptoms
CLEOS: Clinical Expert Operating System
DIAANA AMHTD: Diagnosis and Anamnesis Automated Medical History–Taking Device
EHR: electronic health record
HPI: history of present illness
PGHD: patient-generated health data
PRISMA: Preferred Reporting Items for Systematic Reviews and Meta-Analyses
PRISMA-ScR: Preferred Reporting Items for Systematic Reviews and Meta-Analyses extension for Scoping Reviews
