# Supplementary material for: Digital Tools Designed to Obtain the History of Present Illness From Patients: Scoping Review
Source: J Med Internet Res. 2022 Nov 17;24(11):e36074. doi: 10.2196/36074 (PMC9716422; doi:10.2196/36074)
Supplement: Multimedia Appendix 1 [file jmir_v24i11e36074_app1.docx]

## Multimedia Appendix 1. Search Strategy

Search Terms:

CONCEPT #1: MEDICAL HISTORY / HPI – 29,713 results

“Medical history taking”[tiab] OR “symptom checking”[tiab] OR “symptom checker”[tiab] OR “symptom assessment”[tiab] OR medical history taking[MESH] OR “history of present illness”[tiab] OR HPI[tiab] OR “history-taking”[tiab]

AND

CONCEPT #2: ELECTRONIC INTERFACE – 802,954 results

((Electronic[tiab] OR digital[tiab] OR online[tiab] OR portal[tiab] OR computer*[tiab] OR smart phone[tiab] OR mhealth[tiab] OR ehealth[tiab] OR on-line[tiab] OR interactive technology[tiab] OR kiosk[tiab] OR tablet[tiab] OR “human-computer interface”[tiab]))

AND

CONCEPT #3: PATIENT-GENERATED – 2,160,402 results

((Patient OR patients OR consumer OR people OR user OR person OR persons OR self) AND (generated OR reported OR shared OR facing OR centered OR driven OR administered OR participation OR oriented OR involvement))
