# Supplementary material for: Digital Tools Designed to Obtain the History of Present Illness From Patients: Scoping Review
Source: J Med Internet Res. 2022 Nov 17;24(11):e36074. doi: 10.2196/36074 (PMC9716422; doi:10.2196/36074)
Supplement: Multimedia Appendix 2 [file jmir_v24i11e36074_app2.docx]

## Multimedia Appendix 2. List of Synonyms for digital tools designed to obtain the HPI from patients

## # SYNONYM

## 1 Computer-Administered History

## 2 Automated Medical History

## 3 Automated Patient History Acquisition System

## 4 Patient-Driven Computerized Medical History Export System

## 5 Computerized Medical Interview

## 6 Interactive Computerized Interview

## 7 Computerized History Software

## 8 Patient Directed History

## 9 Online Patient Interview

## 10 Patient-Driven Health Information Technology

## 11 Self-Administered Computer-Assisted Interviewing

## 12 Self-Administered, Automated Medical History Taking Device

## 13 Computer-Generated HPI(s)

## 14 Computerized Collection of Patient Medical History

## 15 Patient Intake & Documentation Tool

## 16 Computer-Assisted Anamnesis

## 17 Computer-Directed, Patient Self-Entry

## 18 Computerized, Patient-Entered Medical Histories
